# Supplementary material for: A morphogenetic EphB/EphrinB code controls hepatopancreatic duct formation
Source: Nat Commun. 2019 Nov 19;10:5220. doi: 10.1038/s41467-019-13149-7 (PMC6864101; doi:10.1038/s41467-019-13149-7)
Supplement: Supplementary file 1 — Supplementary Information [file 41467_2019_13149_MOESM1_ESM.pdf]

SUPPLEMENTARY INFORMATION

A morphogenetic EphB/EphrinB code controls hepatopancreatic duct formation

Thestrup et al.

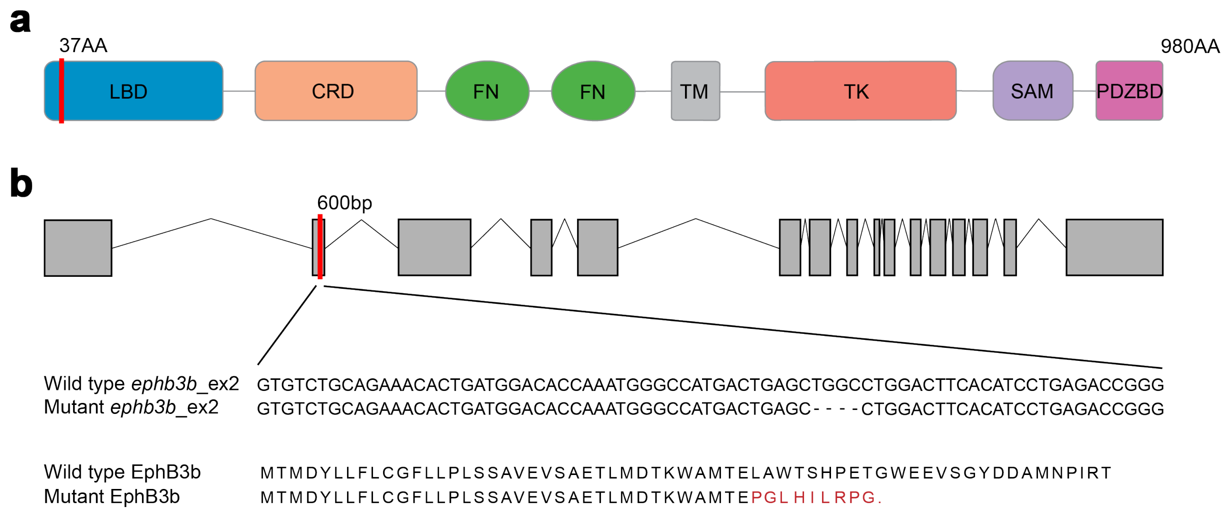

Supplementary Figure 1: Generation of *ephb3b*^{nim27} allele by nuclease-mediated gene editing. a Schematic representation of the EphB3b protein domains. **b** A 4bp deletion was identified in position 600 of *ephb3b* ORF. The nonsense mutation is located in exon 2 of *ephb3b,* one of the exons coding for the ligand-binding domain, truncating the 980aa protein at residue 37 (indicated by red line in **a**). LBD: ligand binding domain; CRD: cysteine-rich domain; FN: fibronectin repeat; TM: transmembrane domain; TK: tyrosine kinase domain; SAM: sterile alpha motif; PDZBD: PDZ binding domain

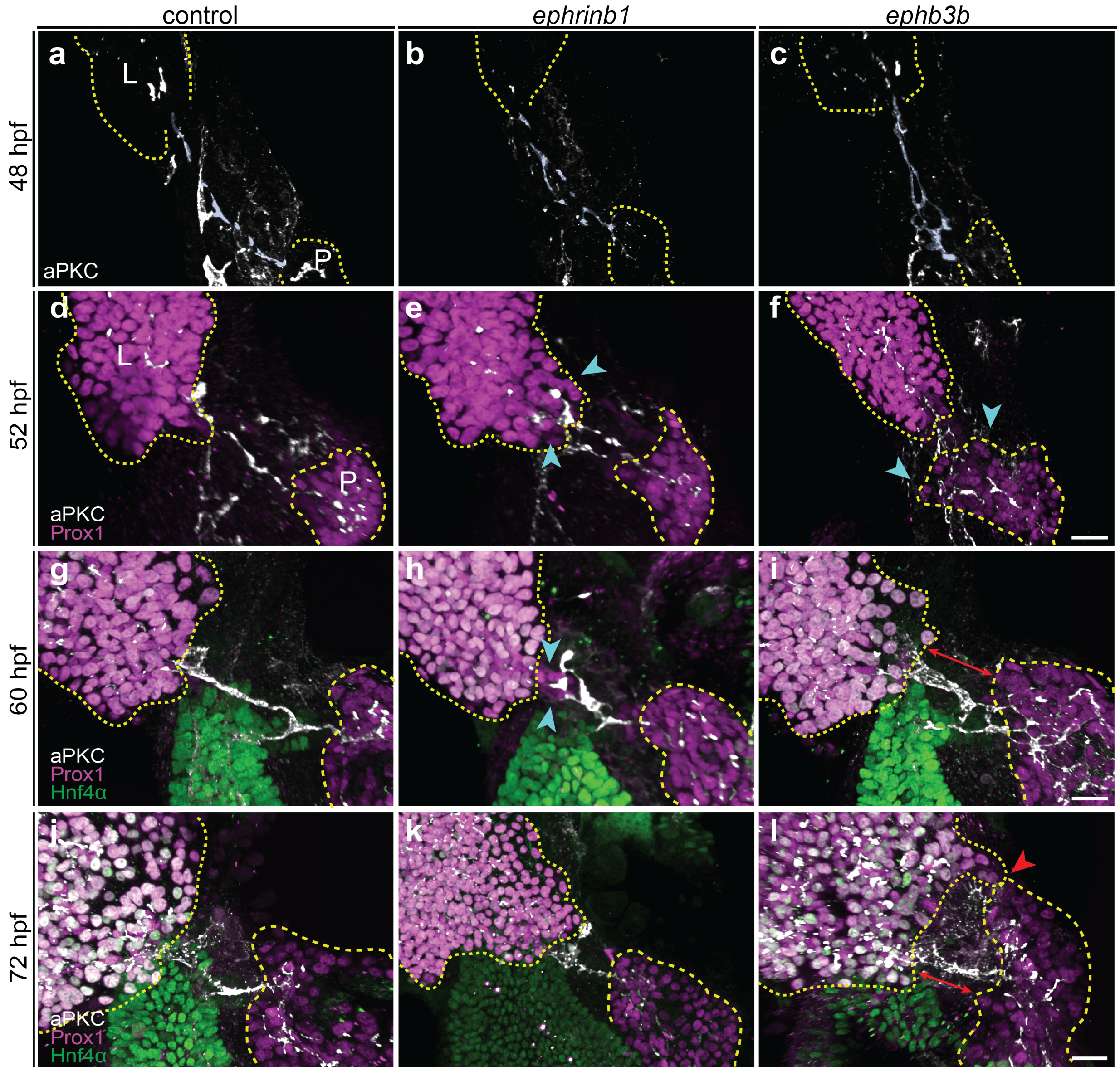

Supplementary Figure 2: Liver and pancreas morphogenesis defects in *ephrinb1* and *ephb3b* mutants. a-c aPKC staining shows initiation of HPD tube formation in controls **(a)**, *ephrinb1* **(b)** and *ephb3b* **(c)** mutant embryos at 48 hpf. Apical aPKC structures in the HPD (light blue) are slightly more complex in both mutants. **d-l** Liver (L) and pancreas (P) progenitors express Prox1 (magenta) between 52 and 72 hpf, while most HPD progenitors have down-regulated Prox1. Differentiating hepatocytes express Prox1 and Hnf4a (green). **e,f** In addition to HPD defects (Fig. 2), both liver and pancreas are dysmorphic in *ephrinb1* and *ephb3b* mutants, and often closer to each other, most notably in *ephb3b* mutants (red arrow and arrowhead in **l**). Ectopic Prox1^{+}/Hnf4α^{-} cells (blue arrowheads) are transiently detected at the proximal base of the liver in *ephrinb1* mutants between 52 and 60 hpf (**e,h**), while not at 72 hpf (**k**). Scale bars=20 μm.

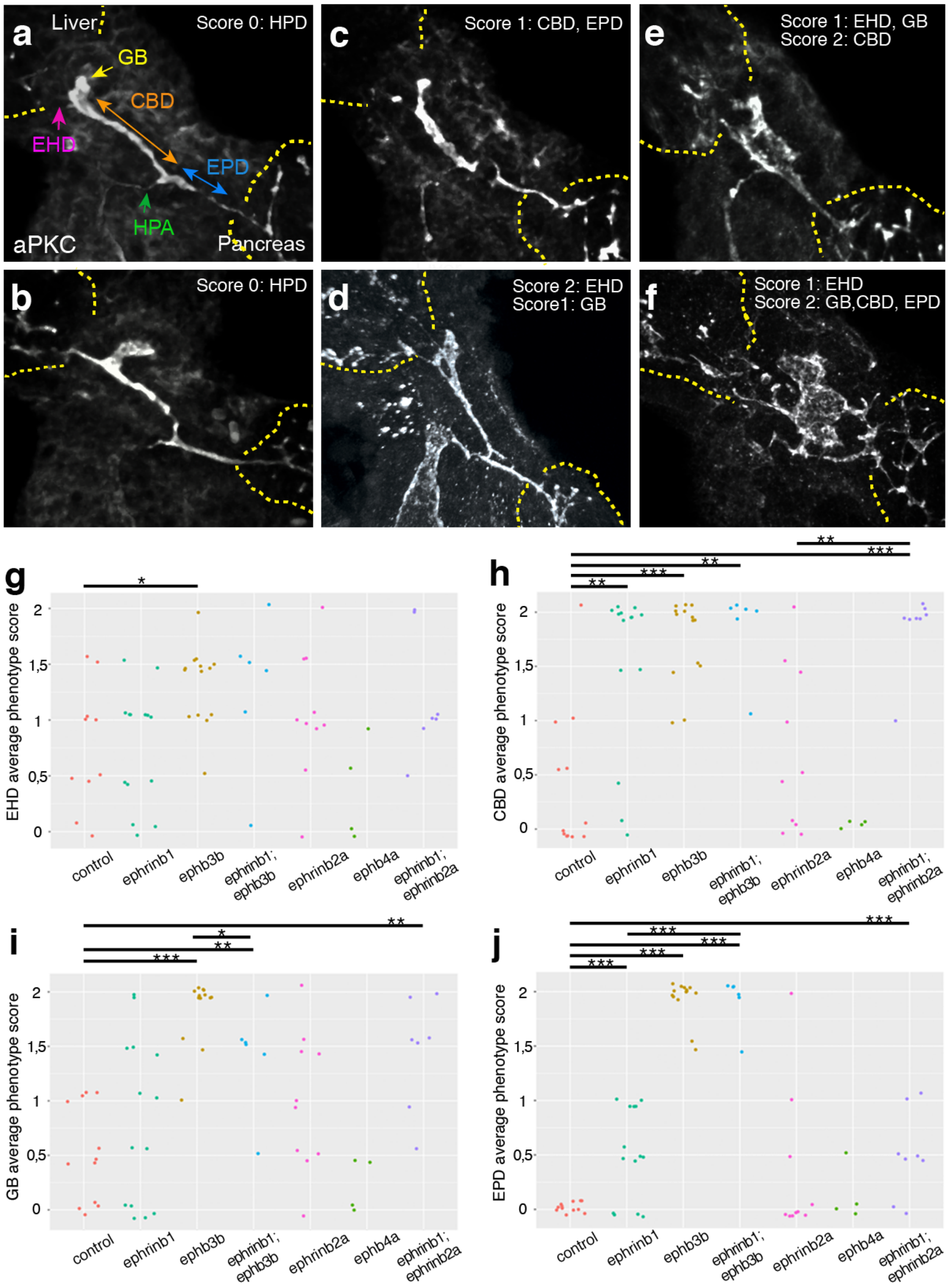

Supplementary Figure 3: Domain-specific analysis of HPD tube morphogenesis. Tube formation is assessed by apical aPKC staining at 60 hpf. **a, b** Embryos with 'no defect' extend a single nascent lumen throughout the HPD system. **c-f** Examples of domain-specific HPD defects categorized as Score1 (=mild) or Score2 (=severe); see Table1. Individual domain scores are indicated in each panel. **g-j** Averaged scoring of two independent assessments of HPD domain defects in control, *ephrinb1, ephb3b, ephrinb1;ephb3b, ephrinb2a, ephb4a* and *ephrinb1;ephrinb2a* embryos are depicted as ordinal values. Frequency data of single scoring result from Figs. 2h, 7f are part of the averaged scores. Statistical test: Wilcoxon non-parametric test; *p<0,05, **p<0,01, ***p<0,001. Source data are provided as a Source Data file. Scale bars = 10 µm.

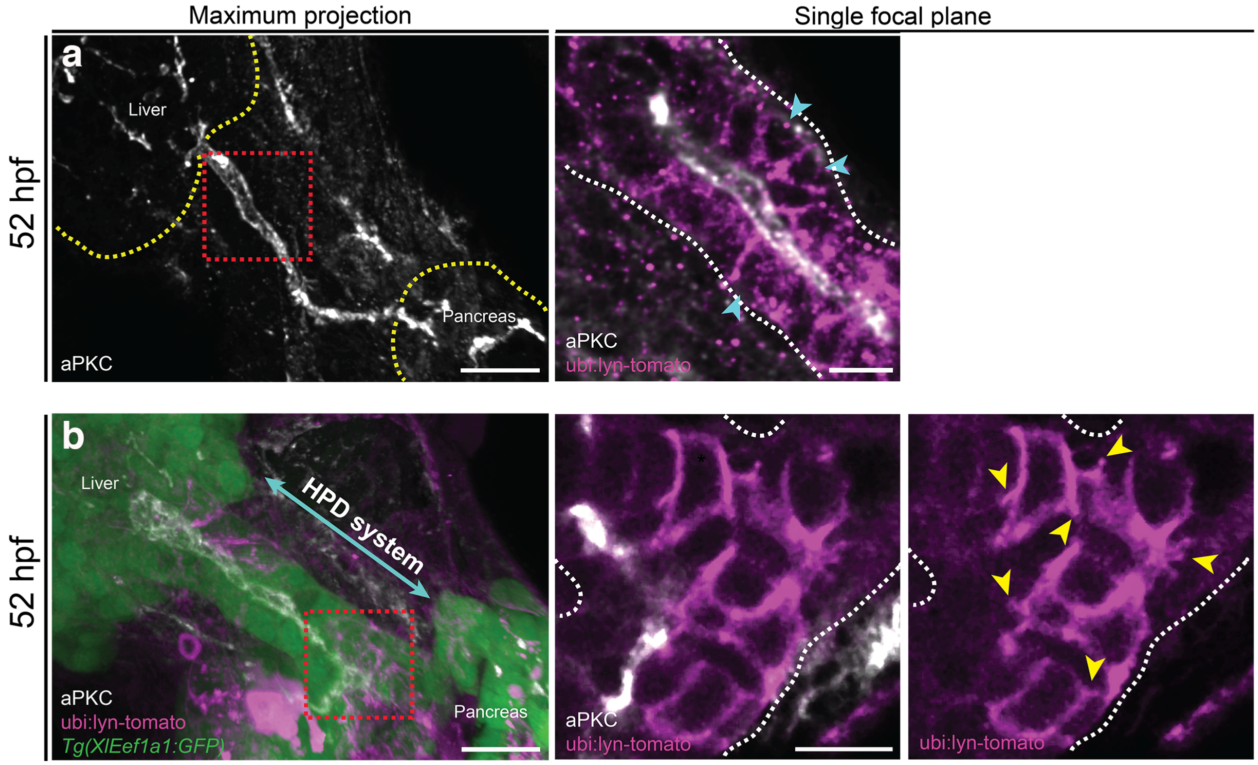

Supplementary Figure 4: CBD cells adopt teardrop shape morphology and extend protrusions during duct remodeling. a PanCadherin staining reveals teardrop-shaped cells (blue arrowheads) in wild-type ductal epithelium at 52 hpf during tube morphogenesis. Single focal plane shows magnified CBD area (red dashed box). **b** Mosaic labeling with membrane tethered Lyn-Tomato visualizes HPD cells form small protrusions (yellow arrowheads). Single focal planes show magnified areas of CDB-EPD junction at the ampulla (red dashed box). Scale bars: maximum projections = 20 μm, single focal planes = 8 μm.

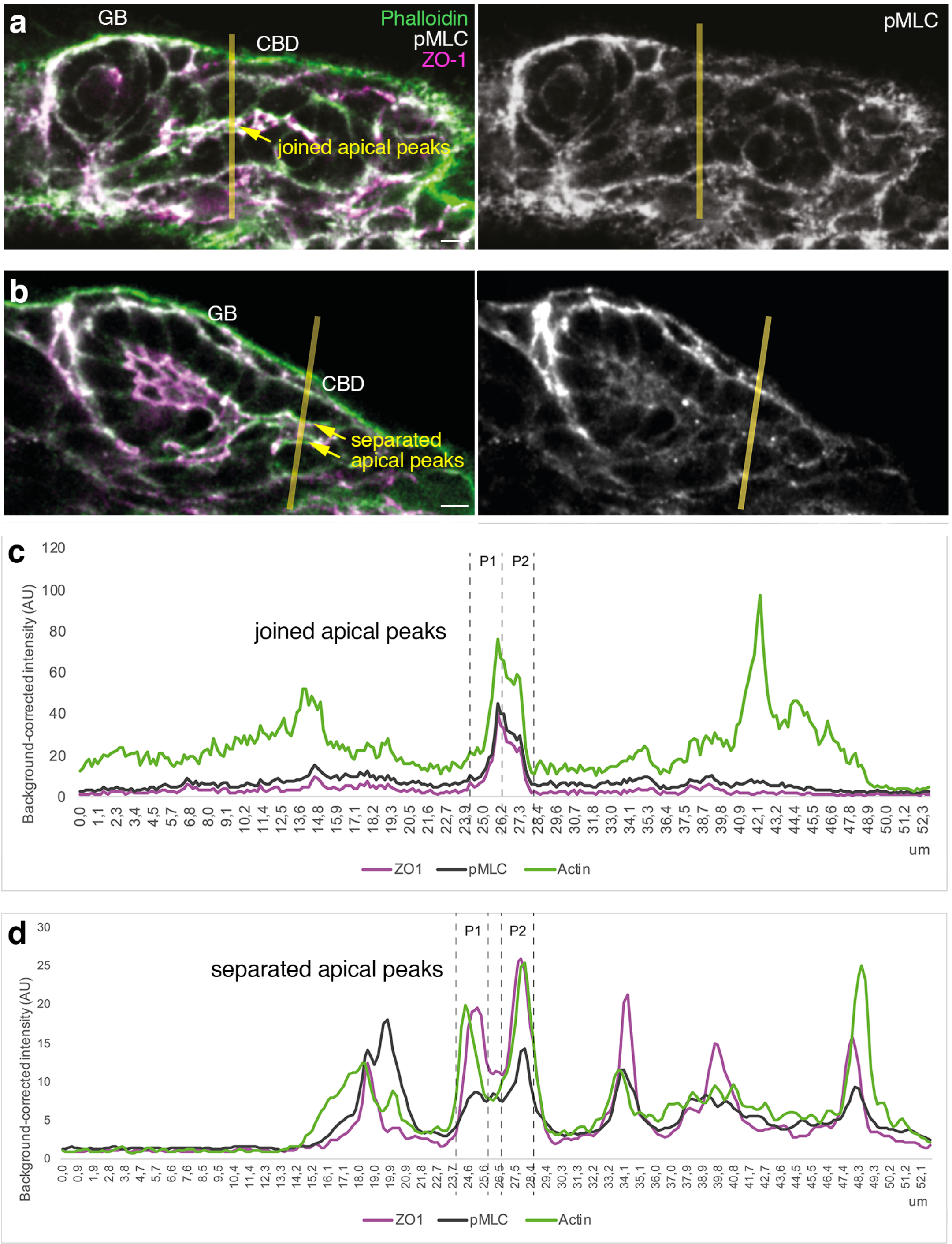

Supplementary Figure 5: Quantification strategy for determining apical pMLC levels. a-b Single focal planes of CBD stained for ZO-1 (magenta), pMLC (white) and actin (green) at 60 hpf depicting 'joined' (**a**) and 'separated' (**b**) apical peaks. **10 pixel wide** ROIs (Region of Interest, yellow lines) perpendicular to the luminal duct axis were used to generate pixel intensity plot profiles of pMLC levels. **c-d** Background-corrected plot profiles of ROIs in CBD, with apical ZO-1 staining aiding peak calling. pMLC intensity values were integrated for 2 μm regions across peaks. **a-b**: Scale bar= 5 μm.

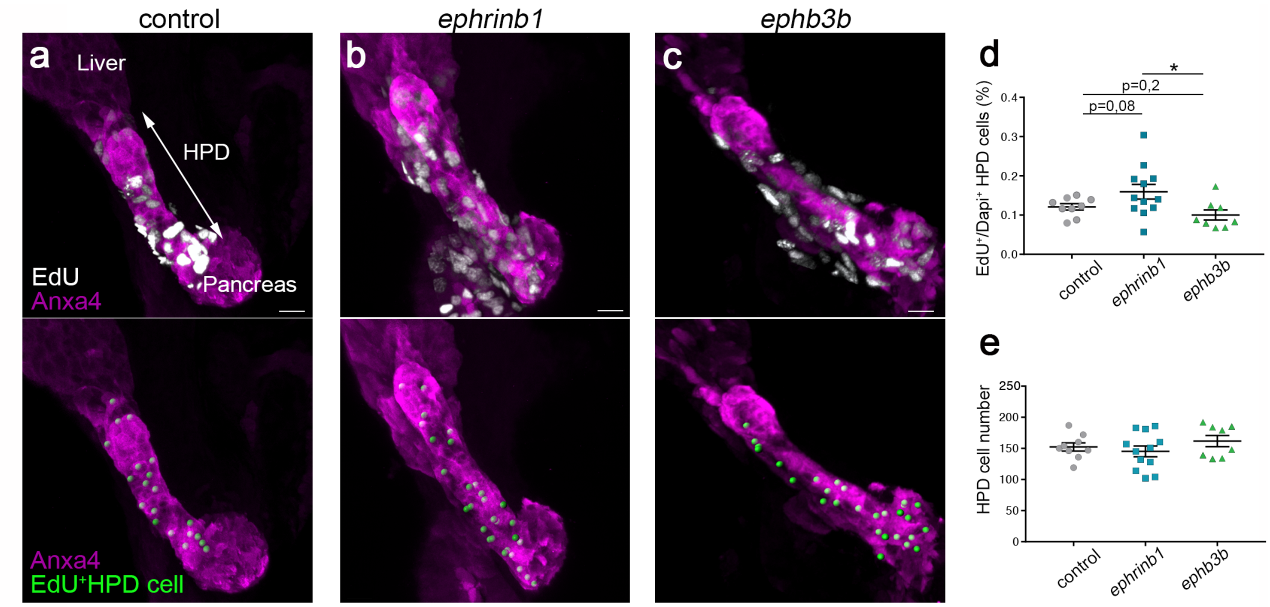

Supplementary Figure 6: Proliferation rates are similar between controls and *ephrinb1* and *ephb3b* mutants during HPD morphogenesis. a-c EdU incorporation between 51-52 hpf highlights proliferating cells in control (**a**, n=9), *ephrinb1* (**b**, n=12) and *ephb3b* (**c**, n=8) embryos. EdU staining (white) is only shown for the approximate area of the HPD using a mask based on ductal Anxa4 expression (magenta). $Edu^+/Dapi^+/Anxa4^+$ cells in the HPD are marked by green spheres for counting. **d, e** Comparison of proliferation rates between genotypes by quantification of $Edu^+/Dapi^+$ cells **(d)** and overall HPD cell numbers **(e)** at 52 hpf. Statistical test: **d, e** = Student's t-test. Error bars show SEM; *p<0,05. Source data are provided as a Source Data file. Scale bar = 10 µm.

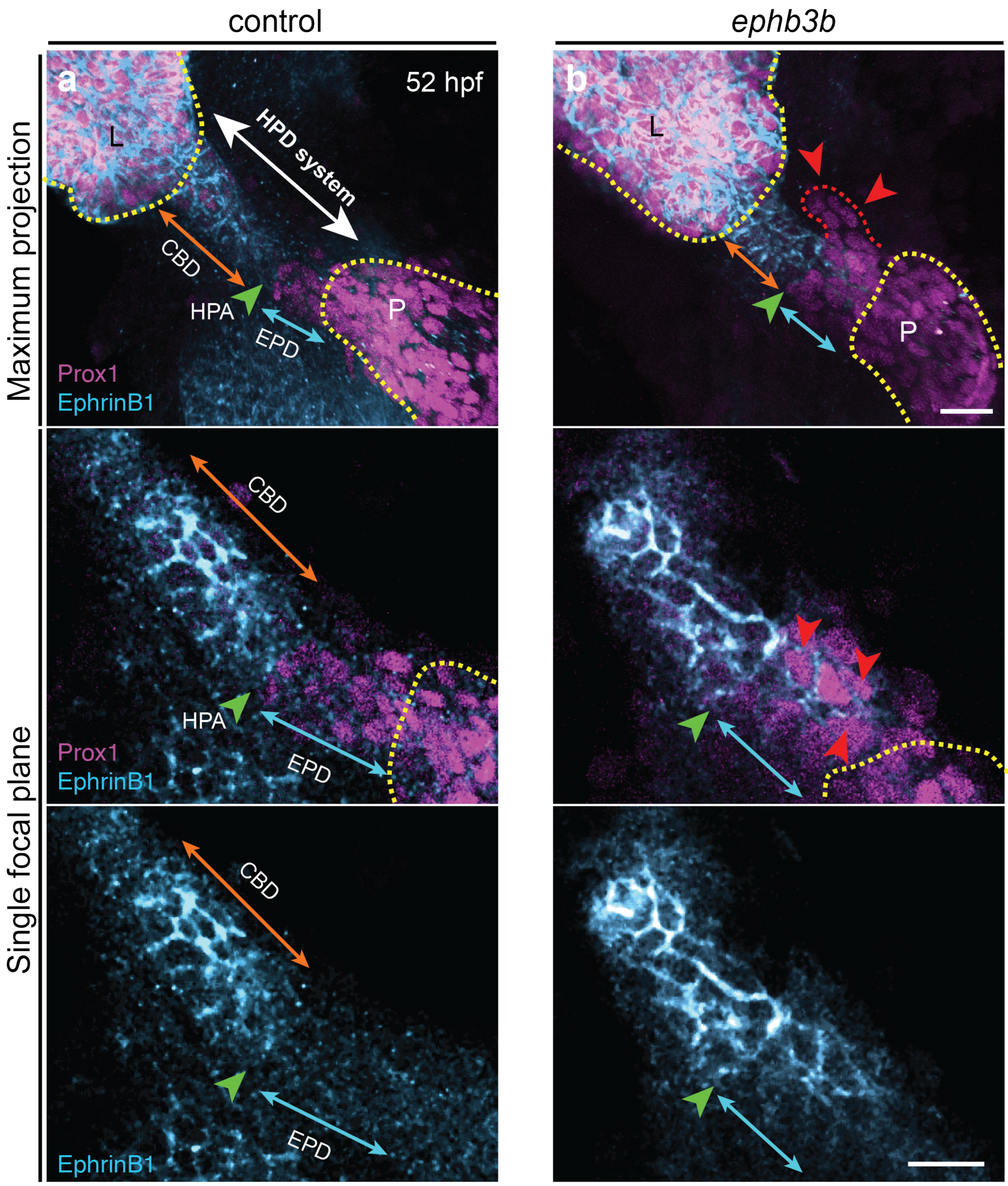

Supplementary Figure 7: Ductal EphrinB1 expression is expanded in *ephb3b* mutants. a EphrinB1 is expressed in the liver and the forming CBD in controls. **b** In *ephb3b* mutants EphrinB1 expression expands from the CBD into the prospective EPD domain, indicated by co-expression with Prox1 (red arrowheads). Ectopic Prox1 (red dashed line) indicates defective pancreas morphology. CBD=orange arrow; EPD=blue arrow; HPA=green arrowhead; scale bar: maximum projections = 20 µm; single focal planes = 15 µm.

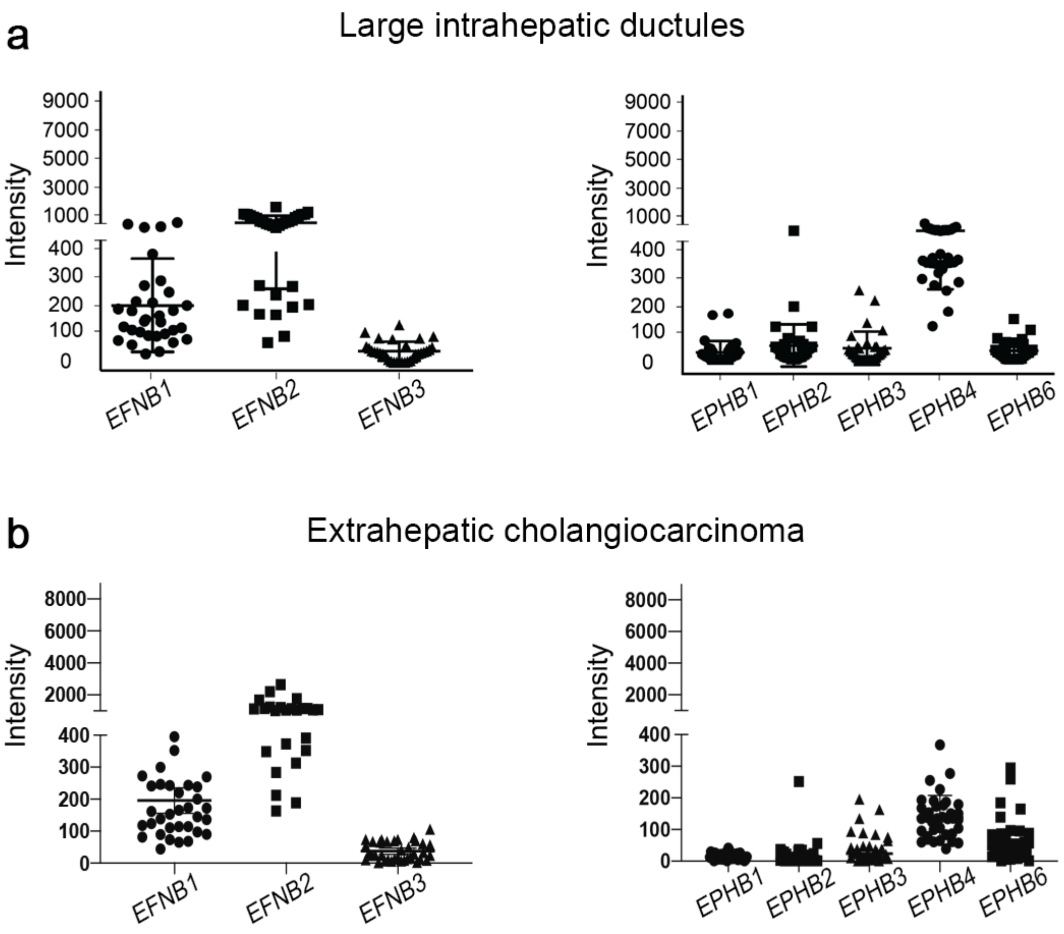

Supplementary Figure 8: EPHRINB ligand and EPHB receptor expression in human large bile ducts and extrahepatic ductal tumor tissue. Microarray analysis shows EPHRINB (EFNB) ligand and EPHB receptor expression in adult human large intrahepatic ductules (**a**) and extrahepatic cholangiocarcinoma (eCCA) tissue (**b**). Error bars show SEM.
