# Supplementary material for: A morphogenetic EphB/EphrinB code controls hepatopancreatic duct formation
Source: Nat Commun. 2019 Nov 19;10:5220. doi: 10.1038/s41467-019-13149-7 (PMC6864101; doi:10.1038/s41467-019-13149-7)
Supplement: Supplementary file 3 — Reporting Summary [file 41467_2019_13149_MOESM3_ESM.pdf]

nature research

Corresponding author(s): Elke Ober

Last updated by author(s): Jul 31, 2019

Reporting Summary

Nature Research wishes to improve the reproducibility of the work that we publish. This form provides structure for consistency and transparency in reporting. For further information on Nature Research policies, see Authors & Referees and the Editorial Policy Checklist.

Please do not complete any field with "not applicable" or n/a. Refer to the help text for what text to use if an item is not relevant to your study.
For final submission: please carefully check your responses for accuracy; you will not be able to make changes later.

Statistics

For all statistical analyses, confirm that the following items are present in the figure legend, table legend, main text, or Methods section.

n/a	Confirmed	
☐	☒	The exact sample size (*n*) for each experimental group/condition, given as a discrete number and unit of measurement
☒	☐	A statement on whether measurements were taken from distinct samples or whether the same sample was measured repeatedly
☐	☒	The statistical test(s) used AND whether they are one- or two-sided<br>*Only common tests should be described solely by name; describe more complex techniques in the Methods section.*
☒	☐	A description of all covariates tested
☐	☒	A description of any assumptions or corrections, such as tests of normality and adjustment for multiple comparisons
☐	☒	A full description of the statistical parameters including central tendency (e.g. means) or other basic estimates (e.g. regression coefficient) AND variation (e.g. standard deviation) or associated estimates of uncertainty (e.g. confidence intervals)
☒	☐	For null hypothesis testing, the test statistic (e.g. *F*, *t*, *r*) with confidence intervals, effect sizes, degrees of freedom and *P* value noted<br>*Give P values as exact values whenever suitable.*
☒	☐	For Bayesian analysis, information on the choice of priors and Markov chain Monte Carlo settings
☒	☐	For hierarchical and complex designs, identification of the appropriate level for tests and full reporting of outcomes
☒	☐	Estimates of effect sizes (e.g. Cohen's *d*, Pearson's *r*), indicating how they were calculated

Our web collection on statistics for biologists contains articles on many of the points above.

Software and code

Policy information about availability of computer code

Data collection	Image acquisition: Zen (black) for Zeiss LSM780 and LSM 880 microscopes and LAS X for Leica SP8 microscope
Data analysis	Image analysis: Imaris (Bitplane), FIJI (Schindelin, J. et al. Nat Meth 9, 676–682 (2012),<br>Statistical Analysis: Microsoft Excel, Prism, R, VasserStats web interface

For manuscripts utilizing custom algorithms or software that are central to the research but not yet described in published literature, software must be made available to editors/reviewers. We strongly encourage code deposition in a community repository (e.g. GitHub). See the Nature Research guidelines for submitting code & software for further information.

Data

Policy information about availability of data

All manuscripts must include a data availability statement. This statement should provide the following information, where applicable:
- Accession codes, unique identifiers, or web links for publicly available datasets
- A list of figures that have associated raw data
- A description of any restrictions on data availability

The image data generated and analyzed in this study are available from the corresponding author upon reasonable request. A source data file for the quantifications shown in Figs. 1g, 2h,o,p, 3e-g, 4e,h, 7f,l,m,p and Supplementary Figs. 3g-j and 6d,e is provided as supplementary data with this manuscript.

Field-specific reporting

Please select the one below that is the best fit for your research. If you are not sure, read the appropriate sections before making your selection.

[X] Life sciences [ ] Behavioural & social sciences [ ] Ecological, evolutionary & environmental sciences

Life sciences study design

All studies must disclose on these points even when the disclosure is negative.

Sample size	Sample sizes were not pre-calculated using statistical tests but following the standard of the field.
Data exclusions	No data were excluded.
Replication	Control and experimental animals were selected from pools of embryos derived from different pair-wise matings at defined developmental stages. Experiments are generally carried out with an N =2 or >2, or otherwise indicated.
Randomization	Quantification of domain-specific HPD defects of controls and all genotypes reported in Figures 2h, 7f and Supplementary Figure 3 were performed by randomizing and blinding all samples, which were subsequently scored independently by two of the authors.
Blinding	Quantification of domain-specific HPD defects of controls and all genotypes reported in Figures 2h, 7f and Supplementary Figure 3 were performed by randomizing and blinding all samples, which were subsequently scored independently by two of the authors.

Reporting for specific materials, systems and methods

We require information from authors about some types of materials, experimental systems and methods used in many studies. Here, indicate whether each material, system or method listed is relevant to your study. If you are not sure if a list item applies to your research, read the appropriate section before selecting a response.

Materials & experimental systems

n/a	Involved in the study	
[ ]	[X]	Antibodies
[X]	[ ]	Eukaryotic cell lines
[X]	[ ]	Palaeontology
[ ]	[X]	Animals and other organisms
[X]	[ ]	Human research participants
[ ]	[X]	Clinical data

Methods

n/a	Involved in the study	
[X]	[ ]	ChIP-seq
[X]	[ ]	Flow cytometry
[X]	[ ]	MRI-based neuroimaging

Antibodies

Antibodies used

mouse α-Prox1 (1:50; Abcam, cat# ab33219, lot#GR296243-3)
rabbit α-Prox1 (1:500; Angiobio, cat# 11-002)
mouse α-2F11 (1:1000; gift from Julian Lewis)
goat α-Hnf4a (1:100; Santa Cruz, cat# sc-6556, lot# B1605)
rabbit aPKCζ (1:1000; Santa Cruz, cat# sc-216, lot# L0105)
mouse α-pan-Cadherin (1:1000; Sigma, cat#CD1821)
mouse α-ZO1 (1:200, Invitrogen, cat#33-9100)
rabbit α-pMLC (Ser19; 1:100; Cell Signaling, cat# #3671)
rabbit α-EphrinB1 (custom-made by Ober group; Cayuso et al. 2016)
guinea pig α-EphB3 (custom-made by Ober group; Cayuso et al. 2016)

Validation

mouse α-Prox1 (1:50; Abcam), identical staining pattern to rabbit α-Prox1 (1:500; Angiobio)
rabbit α-Prox1 (1:500; Angiobio), (Koltowska et al. Cell Rep 2015)
mouse α-2F11 (1:1000; gift from Julian Lewis), (Zhang et al. Dev Biol 2014)
goat α-Hnf4a (1:100; Santa Cruz), (Dong et al. Nat Genetics 2007)
rabbit aPKCζ (1:1000; Santa Cruz), (Horne-Badovinac et al. Curr Biol 2001)
mouse α-pan-Cadherin (1:1000; Sigma), (Dong et al. Nat Genetics 2007)
mouse α-ZO1 (1:200, Invitrogen), (Omori and Malicki, Curr Biol, 2006)
rabbit α-pMLC (Ser19; 1:100; Cell Signaling), (Yabe et al PloS Genetics, 2009)
rabbit α-EphrinB1, validation by staining in loss-of-function mutant (Cayuso et al. Dev Cell 2016)
guinea pig α-EphB3, validation by staining in loss-of-function mutant (Cayuso et al. Dev Cell 2016)

Animals and other organisms

Policy information about studies involving animals; ARRIVE guidelines recommended for reporting animal research

Laboratory animals	Species: zebrafish (Danio rerio) embryos and larvae; adults carriers for breeding<br>Strains: Tg(UAS: ephrinb1EC)nim25 (Cayuso et al. 2016), Tg(hsp70l:Gal4)fci1 (gift from David Wilkinson), ephrinb1 nim26 (Cayuso et al. 2016), ephrinb2a hu3393 and ephb4a hu3378 (Stemple Laboratory, direct submission to ZFIN), ephb3b nim27 (generation described in this study), Tg(keratin18:GFP)p314 (Wilkins et al. 2014) and Tg(Xla.Eef1a1:GFP)s854 (Field et al 2003).
Wild animals	*Provide details on animals observed in or captured in the field; report species, sex and age where possible. Describe how animals were caught and transported and what happened to captive animals after the study (if killed, explain why and describe method; if released, say where and when) OR state that the study did not involve wild animals.*
Field-collected samples	*For laboratory work with field-collected samples, describe all relevant parameters such as housing, maintenance, temperature, photoperiod and end-of-experiment protocol OR state that the study did not involve samples collected from the field.*
Ethics oversight	All experiments were performed according to ethical guidelines approved by the Danish Animal Experiments Inspectorate (Dyreforsøgstilsynet).

Note that full information on the approval of the study protocol must also be provided in the manuscript.

Clinical data

Policy information about clinical studies

All manuscripts should comply with the ICMJE guidelines for publication of clinical research and a completed CONSORT checklist must be included with all submissions.

Clinical trial registration	*Provide the trial registration number from ClinicalTrials.gov or an equivalent agency.*
Study protocol	*Note where the full trial protocol can be accessed OR if not available, explain why.*
Data collection	*Describe the settings and locales of data collection, noting the time periods of recruitment and data collection.*
Outcomes	*Describe how you pre-defined primary and secondary outcome measures and how you assessed these measures.*

This checklist template is licensed under a Creative Commons Attribution 4.0 International License, which permits use, sharing, adaptation, distribution and reproduction in any medium or format, as long as you give appropriate credit to the original author(s) and the source, provide a link to the Creative Commons license, and indicate if changes were made. The images or other third party material in this article are included in the article's Creative Commons license, unless indicated otherwise in a credit line to the material. If material is not included in the article's Creative Commons license and your intended use is not permitted by statutory regulation or exceeds the permitted use, you will need to obtain permission directly from the copyright holder. To view a copy of this license, visit http://creativecommons.org/licenses/by/4.0/
