# Supplementary material for: A morphogenetic EphB/EphrinB code controls hepatopancreatic duct formation
Source: Nat Commun. 2019 Nov 19;10:5220. doi: 10.1038/s41467-019-13149-7 (PMC6864101; doi:10.1038/s41467-019-13149-7)
Supplement: Supplementary file 4 — Description of Additional Supplementary Files [file 41467_2019_13149_MOESM4_ESM.pdf]

Title: Supplementary Movie 1:
Description: Rotation displaying the nascent lumen of the forming HPD system in controls at 60 hpf. Confocal projection of wholemount aPKC staining, starting with a ventral view, anterior to the top. Linked to Figure 1 d, 2 d and 7 a.

Title: Supplementary Movie 2:
Description: Rotation displaying the nascent lumen of the forming HPD in ephrinb1 mutant at 60 hpf. Confocal projection of wholemount aPKC staining, starting with a ventral view, anterior to the top. Linked to Figure 2 e and 7 b.

Title: Supplementary Movie 3:
Description: Rotation displaying the nascent lumen of the forming HPD in ephb3b mutant at 60 hpf. Confocal projection of wholemount aPKC staining, starting with a ventral view, anterior to the top. Linked to Figure 2 f.

Title: Supplementary Movie 4:
Description: Rotation displaying the nascent lumen of the forming HPD in ephrinb1;ephb3b mutant at 60 hpf. Confocal projection of whole-mount aPKC staining, starting with a ventral view, anterior to the top. Linked to Figure 2 g.

Title: Supplementary Movie 5:
Description: Rotation displaying the nascent lumen of the forming HPD in ephrinb2a mutant at 60 hpf. Confocal projection of whole-mount aPKC staining, starting with a ventral view, anterior to the top. Linked to Figure 7 c.

Title: Supplementary Movie 6:
Description: Rotation displaying the nascent lumen of the forming HPD in ephrinb1;ephrinb2a mutant at 60 hpf. Confocal projection of whole-mount aPKC staining, starting with a ventral view, anterior to the top. Linked to Figure 7 d.

Title: Supplementary Movie 7:
Description: Rotation displaying the nascent lumen of the forming HPD in ephb4a mutant at 60 hpf. Confocal projection of wholemount aPKC staining, starting with a ventral view, anterior to the top. Linked to Figure 7 e.

Title: Supplementary Movie 8:
Description: Rotation displaying the differentiated HPD in controls at 5 dpf. Confocal projection of whole-mount Anxa4 staining, starting with a ventral view, anterior to the top. Linked to Figure 1 f, 1h-j, 2 l and 7 g.

Title: Supplementary Movie 9:
Description: Rotation displaying the differentiated HPD of an ephrinb1 mutant at 5 dpf. Confocal projection of whole-mount Anxa4 staining, starting with a ventral view, anterior to the top. Linked to Figure 2 m and 7 h.

Title: Supplementary Movie 10:
Description: Rotation displaying the differentiated HPD of an ephb3b mutant at 5 dpf. Confocal

projection of whole-mount Anxa4 staining, starting with a ventral view, anterior to the top. Linked to Figure 2 n.

Title: Supplementary Movie 11:
Description: Rotation displaying the differentia PAGE 5 OF ted HPD of an ephrinb2a mutant at 5 dpf. Confocal projection of whole-mount Anxa4 staining, starting with a ventral view, anterior to the top. Linked to Figure 7 i.

Title: Supplementary Movie 12:
Description: Rotation displaying the differentiated HPD of an ephrinb1;ephrinb2a mutant at 5 dpf. Confocal projection of wholemount Anxa4 staining, starting with a ventral view, anterior to the top. Linked to Figure 7 j.

Title: Supplementary Movie 13:
Description: Rotation displaying the differentiated HPD of an ephb4a mutant at 5 dpf. Confocal projection of whole-mount Anxa4 staining, starting with a ventral view, anterior to the top. Linked to Figure 7 k.
